# Induced Tissue-Specific Stem Cells and Epigenetic Memory in Induced Pluripotent Stem Cells

**DOI:** 10.3390/ijms19040930

**Published:** 2018-03-21

**Authors:** Hirofumi Noguchi, Chika Miyagi-Shiohira, Yoshiki Nakashima

**Affiliations:** Department of Regenerative Medicine, Graduate School of Medicine, University of the Ryukyus, Okinawa 903-0215, Japan; chika@med.u-ryukyu.ac.jp (C.M.-S.); nakasima@med.u-ryukyu.ac.jp (Y.N.)

**Keywords:** induced tissue-specific stem (iTS) cells, induced pluripotent stem (iPS) cells, reprogramming factors, epigenetic memory

## Abstract

Induced pluripotent stem (iPS) cells have significant implications for overcoming most of the ethical issues associated with embryonic stem (ES) cells. The pattern of expressed genes, DNA methylation, and covalent histone modifications in iPS cells are very similar to those in ES cells. However, it has recently been shown that, following the reprogramming of mouse/human iPS cells, epigenetic memory is inherited from the parental cells. These findings suggest that the phenotype of iPS cells may be influenced by their cells of origin and that their skewed differentiation potential may prove useful in the generation of differentiated cell types that are currently difficult to produce from ES/iPS cells for the treatment of human diseases. Our recent study demonstrated the generation of induced tissue-specific stem (iTS) cells by transient overexpression of the reprogramming factors combined with tissue-specific selection. iTS cells are cells that inherit numerous components of epigenetic memory from donor tissue and acquire self-renewal potential. This review describes the “epigenetic memory” phenomenon in iPS and iTS cells and the possible clinical applications of these stem cells.

## 1. Introduction

Embryonic stem (ES) cells and induced pluripotent stem (iPS) cells are capable of unlimited proliferation in vitro while maintaining their potential to differentiate into cells from the three embryonic germ layers [1,2,3,4,5,6,7]. iPS cells have been generated by reprogramming factors such as Oct4, Sox2, Klf4, c-Myc, L-Myc, Nanog, Lin28, SV40LT, p53 shRNA, and/or Glis1 (all mentioned factors are part of different reprogramming cocktails) [1,2,3,4,5,6,7,8,9,10,11,12]. The generation of iPS cells without the genomic integration of exogenous reprogramming factors by plasmids [8,9,10], adenoviruses [11], and RNA [12] has been reported. The production of iPS cells without insertional mutagenesis addresses a critical safety concern regarding the potential use of iPS cells in regenerative medicine.

Recently, we have focused on developing a method for generating induced tissue-specific stem (iTS) cells by transfection with a plasmid harboring cDNAs for Oct3/4, Sox2, Klf4, and c-Myc and subsequent tissue-specific selection [13,14]. The iTS cells were unable to generate teratomas when transplanted subcutaneously into immunodeficient mice. The iTS cells from pancreatic tissue (iTS-P cells) and liver cells (iTS-L cells) expressed several genetic markers for endoderm and pancreatic/hepatic progenitors and differentiated into insulin-producing cells/hepatocytes more efficiently than ES cells upon differentiation induction [13]. Since it was shown that the epigenetic memory predisposes iPS cells derived from pancreatic β-cells to differentiate more readily into insulin-producing cells [15], the iTS-P/iTS-L cells must be cells that inherit numerous components of epigenetic memory from pancreas/liver cells and acquire self-renewal potential.

In the present review, we focus on iTS cells and epigenetic memory in iPS cells.

## 2. Generation of iPS Cells

Takahashi and Yamanaka first reported iPS cells from mouse fibroblast in 2006 [1]. They identified a suite of embryonic transcription factors whose overexpression restored pluripotency to adult somatic cells. Mouse fibroblasts that harbored a drug selection cassette under the control of a promoter active only in ES cells (*Fbx15*) were generated. To induce reprogramming, they transduced mouse embryonic fibroblasts (MEFs) with retroviral vectors encoding a total of 24 genes previously implicated in the biology of ES cells. Although the transduction of any of these factors alone was insufficient to induce the expression of ES markers, simultaneous transduction with all 24 genes followed by antibiotic selection resulted in the appearance of drug-resistant colonies with a morphology similar to that of ES cells. To identify the most important factors among these 24 genes, they repeated the experiment with retroviruses that lacked just 1 of the 24 candidate genes. They selected a set of 10 genes capable of inducing the formation of ES-like morphology. The ES-like cells were termed “iPS-MEF10” cells. By repeating this approach, they ultimately narrowed down the pool of genes required to generate iPS cells. They identified four factors: Oct3/4, Sox2, Klf4, and c-Myc. The “iPS-MEF4” cells had morphological and growth characteristics similar to “iPS-MEF10” cells. They were also able to generate iPS cells from tail-tip fibroblasts from adult mice using these four factors. The “iPS-TTF” cells contributed widely to diverse tissues in chimeric embryos recovered as late as embryonic day 13.5 when the cells were injected into mouse blastocysts.

However, their data also suggested that iPS cells selected by “*Fbx15*” were similar but not identical to ES cells. The expression patterns of ES-specific markers were “clone-dependent”, and only a few iPS clones were similar to ES cells. DNA methylation of the Oct3/4 promoter and the posttranslational modification of histones positioned there suggested that the iPS cells were caught in an epigenetic state that was intermediate between the somatic origins and ES cells. Furthermore, the iPS cells failed to produce adult chimeras. Although they used mouse fibroblasts that were generated harboring a drug selection cassette under the control of *Fbx15*, there was a substantial degree of clone-to-clone variation in the Fbx15-selected iPS cells.

**Figure 1:**
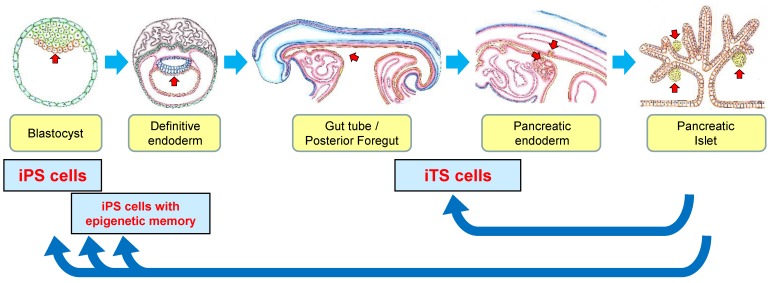
Differentiation of pancreatic islets and generation of iPS/iTS cells. iPS cells have been generated by reprogramming the factors such as Oct4, Sox2, Klf4, and c-Myc. While iPS cells have been shown to be similar to ES cells, several articles have suggested that, following the reprogramming of iPS cells, epigenetic memory is inherited from the parental cells. iTS cells have been generated by the reprogramming factors combined with tissue-specific selection. iTS cells are “incompletely reprogrammed” cells that inherit numerous components of epigenetic memory from donor tissue. Red allows show endodermal cells and pancreatic tissue.

Yamanaka’s group next showed the generation of iPS cells with higher expression of ES-specific markers and ES-like pattern of DNA methylation via the selection for *Nanog* expression instead of *Fbx15* selection [6]. The four reprogramming factors (Oct3/4, Sox2, Klf4, and c-Myc) and *Nanog* selection resulted in germline-competent iPS cells. This report clearly showed that they generated “complete” iPS cells with germline transmission, and the selection of the clones was important for the iPS cells. In other words, the transduction of the four reprogramming factors into somatic cells induced “complete” iPS cells identical to ES cells and “incomplete” iPS cells with epigenetic memory from donor tissue (Figure 1).

Retroviral integration of the transcription factors may activate or inactivate host genes, resulting in tumorigenicity, as was the case in some patients who underwent gene therapy. The second report of Yamanaka’s group [6] included the extremely important finding that, in Nanog-selected iPS cells, the four transgenes (Oct3/4, Sox2, Klf4, and c-Myc) were strongly silenced and endogenous Oct3/4, Sox2, Klf4, and c-Myc were expressed. The data strongly suggested that the transient expression of these four exogenous factors might be sufficient for the generation of iPS cells. In fact, the generation of mouse iPS cells by repeated transfection of plasmids expressing Oct3/4, Sox2, Klf4 and c-Myc [8] and by using nonintegrating adenoviruses transiently expressing the four factors [11] has been reported. These reports provide strong evidence that insertional mutagenesis is not required for in vitro reprogramming.

Human iPS cells were generated from adult somatic cells by introducing Oct3/4 and Sox2 with either (1) Klf4 and c-Myc [2] or (2) Nanog and Lin28 [3] using retroviruses in 2007. Human iPS cells are also similar to human ES cells in their morphology, gene expression, and in vitro differentiation. Furthermore, the generation of human iPS cells without genomic integration of exogenous reprogramming factors by plasmids expressing OCT3/4, SOX2, KLF4, c-MYC, NANOG, LIN28, and SV40LT [10] has been shown. Yamanaka’s group showed a more efficient method of generating integration-free human iPS cells using episomal plasmid vectors expressing OCT3/4, p53 shRNA, SOX2, KLF4, L-MYC, and LIN28 [9]. The administration of synthetic mRNA encoding OCT3/4 SOX2, KLF4, and c-MYC was also shown to reprogram human somatic cells to pluripotency [16]. Recently, a single, synthetic, self-replicating VEE-RF RNA replicon expressing four reprogramming factors (OCT4, KLF4, SOX2, and GLIS1) at consistently high levels prior to regulated RNA degradation was utilized to generate iPS cells [12]. The production of iPS cells without insertional mutagenesis addresses a critical safety concern regarding the potential use of iPS cells in regenerative medicine.

## 3. Properties of iPS Cells Imbued by Epigenetic Memory

While iPS cells have been shown to be similar to ES cells, several articles have suggested that iPS cells differ from ES cells in their gene expression profiles [17], persistence of donor-cell gene expression [18,19], and differentiation abilities [20,21]. It has been reported that, following the reprogramming of iPS cells, epigenetic memory is inherited from the parental cells [22,23,24,25,26]. Kim et al. [22] analyzed ES cells and iPS cells derived from two different somatic cell types: mouse bone marrow cells (Kit^+^, Lin^−^, CD45^+^) and dermal fibroblasts. Blood-derived iPS cells differentiated into hematopoietic colonies more easily than fibroblast-derived iPS cells. In contrast, fibroblast-derived iPS cells differentiated into osteoblasts and showed higher expression of osteoblast-associated genes than blood-derived iPS cells. Other groups showed the differentiation potentials of human iPS cells from neonatal umbilical blood cells and foreskin keratinocytes [27]. The expression of an early differentiation marker, the keratin-14 gene, was 9.4-fold higher in iPS cells derived from keratinocytes than those derived from the umbilical blood, indicating a much higher differentiation potential for iPS cells from keratinocytes towards keratinocytes than for iPS cells from the umbilical blood. In contrast, the differentiation potential of iPS cells from umbilical blood to hematopoiesis was markedly increased compared with keratinocytes. Bar-Nur et al. [15] generated human iPS cell lines from pancreatic islet beta cells. Although beta cell-derived iPS cells differentiated into the three embryonic germ layers, the iPS cells maintained an open chromatin structure at key beta cell genes, along with a unique DNA methylation signature that distinguishes them from ES cells and iPS cells derived from other cell types. Beta cell-derived iPS cells differentiated more readily into insulin-producing cells both in vitro and in vivo, compared with ES cells.

Stadtfeld et al. [28] examined several murine iPS cells derived from hematopoietic stem cells (11 lines), granulocyte-macrophage progenitor (11 lines), granulocytes (nine lines), peritoneal fibroblasts (six lines), tail-tip fibroblasts (six lines), and keratinocytes (six lines). The overall messenger RNA and microRNA expression patterns were indistinguishable with the exception of a few transcripts encoded within the imprinted *Dlk1-Dio3* gene cluster on chromosome 12qF1, which were aberrantly silenced in most of the iPS cell clones. These iPS cell clones contributed poorly to chimaeras and failed to support the development of entirely iPS cell-derived animals. In contrast, iPS cell clones with a normal expression of the *Dlk1-Dio3* cluster contributed to high-grade chimeras and generated viable, entirely iPS cell-derived animals. These data suggest that some iPS cell clones are markedly similar to ES cells and that other iPS cell clones differ from ES cells with regard to their contribution to chimeras and the development of entirely ES/iPS cell-derived animals.

## 4. DNA Methylation in iPS Cells

ES cells and iPS cells share similar gene expression profiles, histone covalent modifications, DNA methylation, and microRNA expression. However, there are slight differences between ES and iPS cells and among independent iPS cell lines in their transcriptomes and epigenomes. The different patterns of DNA methylation in independent iPS cell lines have been analyzed in a number of recent studies. Kim et al. [22] evaluated the methylation of approximately 4.6 million CpG nucleotides, including virtually all CpG islands and the adjacent areas while ignoring non-CpG methylation. A total of 3349 differentially methylated regions (DMRs) were found in fibroblast-derived iPS cells, and 516 DMRs were found in blood-derived iPS cells compared with ES cells. A total of 5202 DMRs were identified between blood-derived iPS cells and fibroblast-derived iPS cells. Furthermore, genes for the top 24 DMRs that distinguish blood-derived iPS cells and fibroblast-derived iPS cells linked 11 to hematopoiesis and 3 to osteogenesis. These results suggest that these iPS cells have epigenetic memory. Polo et al. [23] demonstrated that iPS cells derived from mouse fibroblasts, hematopoietic and myogenic cells exhibited distinct transcriptional and epigenetic patterns and that their cellular origin influenced the in vitro differentiation potentials of iPS cells into embryonic bodies and different hematopoietic cell types.

A similar phenomenon has also been reported in human iPS cells. Nishino et al. [29] analyzed the DNA methylation profiles of 22 human iPS cell lines derived from five different cell types (endometrium, placental artery endothelium, amnion, fetal lung fibroblast, and menstrual blood cell), five human ES cell lines, and six lines of initial somatic cells. About 90% of the CpG sites (17,572 sites) showed no markedly different methylation among iPS, ES, and initial somatic cells. A total of 174 sites (79.5%) of 220 pluripotent stem-cell-specific DMRs had significantly higher methylation levels in iPS/ES cells than in initial somatic cells. For the whole genome, the number of DMRs varied between ES cells and iPS cells in 22 iPS cell lines. A total of 1459 DMRs covering 1260 genes were found to be differentially methylated across multiple iPS cell lines. The study found a significant effect of long term culture on differences between ES and iPS cells. The longer the iPS cell lines have been kept in culture the closer their methylation profiles became to bona fide ES cells. Other groups have described the DNA methylation in human ES cells and iPS cells from neonatal umbilical blood (from two independent donors) [27]. Of 370 DMRs in ES cells and iPS cells, 267 were acquired *de novo* due to reprogramming, while 75 were inherited via epigenetic memory. Lister et al. [24] used the highly sensitive MethylC-Seq method to compare the methylomes of several iPS cell lines derived from somatic cells of various types using various approaches. A total of 1175 DMRs between ES and iPS cells were detected. Other groups also described the DNA methylation profiles in iPS cells derived from several somatic cells [30,31]. These studies have shown the presence of minimal differences in the patterns of DNA methylation between ES cells and iPS cells. The DMRs inherited through epigenetic memory cause the iPS cell lines to differentiate into somatic cells of the initial type.

Bar-Nur et al. [15] evaluated the levels of histone H3 acethylation, a hallmark of open chromatin structure, by chromatin immunoprecipitation (ChIP) in iPS cells derived from human pancreatic β-cells. The β-cells-derived iPS cells maintain a partially open chromatin structure on the promoter regions of *INSULIN* and *PDX1*, as judged by histone H3 acetylation. In contrast, the histon H3 on the *MAFA* promoter underwent deacetylation during reprogramming into iPS cells. Stadtfeld et al. [28] showed that the acetylation levels of the H3 and H4 histones and that of methylated *H3K4* associated with transcriptionally active chromatin are significantly lower in *Dlk1-Dio3* locus of iPS cells poorly to chimaeras.

## 5. Tissue-Specific Stem Cells

Since damaged tissues are repaired even in elderly people, adult tissue-specific stem/progenitor cells seem to be present and active within the body throughout life. Tissue-specific stem/progenitor cells play central roles in the maintenance, repair, and reconstitution of tissues, regulated by homeostatic and regenerative signals [32]. Tissue-specific stem cells were initially identified in tissues with a high turnover, namely the skin and gut [33]. More recently, adult stem cells have also been identified in tissues with a low regeneration potential, such as the brain [34], kidney [35], and pancreas [36,37,38,39,40,41,42,43,44,45,46,47,48,49].

Pancreatic islet neogenesis, the budding of new islets from pancreatic stem/progenitor cells located in or near ducts, has long been assumed to be an active process in the postnatal pancreas. Several in vitro studies have shown that insulin-producing cells can be generated from adult pancreatic ductal tissues [38,39,40,41,42,43,44]. Seaberg et al. investigated the clonal source of pancreatic stem/progenitor cells using a serum-free, colony-forming assay [45]. The identified multipotent precursor cells proliferate in vitro to form clonal colonies that co-express neural and pancreatic precursor markers. These pancreas-derived cells appear to have a limited capacity for self-renewal, lack the ES cell markers Oct4 and Nanog, and are of neither mesodermal nor neural crest origin. Upon differentiation, individual clonal colonies produce distinct populations of neurons and glial cells: pancreatic endocrine β, α, and δ cells; and pancreatic exocrine and stellate cells. Pancreas-related proteins are expressed in 4−6% of these cells.

On the other hand, it is not easy to isolate pancreatic “stem” cells, which have a self-renewal capacity. We and other groups have isolated mouse pancreatic stem cell lines using specific culture conditions [36,46]. One of our isolated pancreatic stem cell lines, HN#13, derived from the pancreatic tissue of an eight-week-old mouse without genetic manipulation, can be differentiated into insulin-producing cells and maintained during repeated passages for more than one year without growth inhibition under specific culture conditions. The mouse pancreatic stem cells do not have tumorigenic properties and have normal chromosomes. The cells express pancreatic and duodenal homeobox factor-1 (Pdx1), a transcription factor of the β cell lineage [46]. However, we have been unable to isolate and culture mouse pancreatic stem cells from older donors [47] or human pancreatic stem cells from adult pancreatic tissue [48]. In a mouse study, pancreatic stem cells were isolated from the pancreata of all newborn mice examined but from only 10% of the pancreata of 8-week-old mice and from no pancreata of any 24-week-old mice [47]. These data suggest that young donors have a larger number of pancreatic stem cells than older donors and that it is extremely difficult to isolate pancreatic stem cells from older donors.

## 6. Induced Tissue-Specific Stem Cells

Recently, our group demonstrated the generation of iTS cells by the transient overexpression of reprogramming factors using a plasmid combined with tissue-specific selection. The plasmid transfection into mouse pancreatic tissue resulted in the efficient generation of iTS cells from pancreas (iTS-P cells) with genetic markers of endoderm and pancreatic progenitors, such as Pdx1, and differentiation into insulin-producing cells more efficiently than ES cells. Subcutaneous transplantation of iTS-P cells into immunodeficient mice resulted in no teratoma formation. We next attempted to achieve the efficient selection of iTS-P cells, since there was a large number of fibroblast-like colonies also included in the above experiments. Since iTS-P cells expressed the Pdx1 transcription factor at both the mRNA and protein level, we used a plasmid containing a neomycin-resistance (NeoR) gene that was driven by the Pdx1 promoter. We transfected the reprogramming plasmid and pPdx1-NeoR plasmid together into the mouse pancreatic tissue on days 1, 3, 5 and 7, and G418 was added to the ES culture media from day 10 to 15 in order to select Pdx1-expressing cells. We obtained multiple iTS-P colonies that had self-renewal capacity and were morphologically similar to pancreatic stem cells. There were no fibroblast-like colonies in this experiment [13,14]. ES/iPS cells carry a risk of teratoma formation, even after transplantation of differentiated cells derived from ES/iPS cells, due to possible contamination with undifferentiated cells. This is one of the advantages of iTS-P cells over ES/iPS cells in terms of potential clinical use.

Bisulfite genomic sequencing in iTS-P cells demonstrated that the promoters of Oct3/4 and Nanog remained methylated in iTS-P cells. Furthermore, quantitative reverse transcription polymerase chain reaction (qRT-PCR) showed that there were few expressions of Oct3/4 or Nanog (unpublished data). These results suggest that methylation of the promoters in iTS-P cells is not similar to that in ES cells; therefore, iTS-P cells are unlikely to have pluripotency or teratoma formation. We also evaluated the global gene-expression profiles of ES cells, iTS-P cells, and pancreatic islets using microarrays. The microarray data showed that iTS-P cells were markedly different from iPS cells and pancreatic islets. Of note, the percentage of shared expression genes were higher in both ES cells and iTS-P cells than in iTS-P cells and pancreatic islets [unpublished data], suggesting that iTS-P cells were more closely related to ES cells than pancreatic islets. Our group also generated iTS cells from liver cells (iTS-L cells) [13] and from mesenchymal cells (iTS-M cells) (unpublished data) via the transient overexpression of the reprogramming factors combined with tissue-specific selection. The data of bisulfite genomic sequencing and microarrays of iTS-P cells and the evidence of iTS generation from several tissues suggest that iTS cells are “incompletely reprogrammed” cells that inherit numerous components of epigenetic memory from donor tissue (Figure 1).

Nakajima-Koyama et al. investigated whether or not astrocytes are reprogrammed into iPS cells through a neural stem cell (NSC)-like state [50]. They generated iPS cells from mouse astrocytes by two (OCT3/4 and KLF4), three (OCT3/4, KLF4, and SOX2), or four (OCT3/4, KLF4, SOX2, and c-MYC) factors. Sox1, 2, and 3 transcription factors are important for NSC maintenance because they inhibit differentiation. Since the expression of Sox1 is limited to NSCs, they used Sox1 as an NSC-specific marker. Sox1 is transiently up-regulated during reprogramming, and Sox1-positive cells become iPS cells. The upregulation of Sox1 is essential for OCT3/4- and KLF4-induced reprogramming. A genome-wide analysis revealed that the gene expression profile of Sox1-expressing intermediate-state cells resembles that of NSCs. Furthermore, the intermediate-state cells are able to generate neurospheres, which can differentiate into both neurons and glia cells. Their data suggest that astrocytes are reprogrammed through a transient Sox1-positive state that exhibits some NSC characteristics and that the Sox1-expressing intermediate-state cells may become induced NSCs when the expression of reprogramming factors is off in that state.

Another group recently showed the induction of expandable tissue-specific stem/progenitor cells through the transient expression of YAP/TAZ [51]. They generated mammary, neural, and pancreatic iTS cells by YAP/TAZ. Since YAP/TAZ is essential for organ regrowth after tissue damage or oncogenic transformation in several tissues, such as the liver, pancreas, intestine, and mammary gland [52,53], the generation of iTS cells by YAP/TAZ may suggest potential applications for the development of new treatments for disease.

## 7. Epigenetic Variation between iPS Cell Lines

It was recently reported that epigenetic variation between human iPS cell lines was an indicator of differentiation capacity [54]. Thirty-five human iPS cell lines, including iPS cell lines generated by Yamanaka’s group, and four ES cell lines were used to analyze the relationship of the hematopoietic differentiation performance with molecular signatures, such as the gene expression, DNA methylation, and chromatin status. The hematopoietic commitment of ES and iPS cells to hematopoietic precursors was found to correlate with the insulin-like growth factor 2 (IGF2) expression. This correlation was surprising, as IGF2 itself is not a gene directly related to the hematopoietic lineage, but its expression turns on signaling-dependent chromatin accessibility at genes that are directly related. The maturation capacity for conversion of ES and iPS cell-derived hematopoietic precursors to mature blood is associated with the amount and pattern of DNA methylation acquired during reprogramming. Although this report offers a negative opinion regarding the epigenetic memory of iPS cells, the findings showed that there is epigenetic variation between iPS cell lines and that this variation affects the differentiation of iPS cells.

Another group compared transcriptomic, epigenetic, and differentiation propensities of genetically matched human iPS cells derived from fibroblasts and blood and reprogrammed using the Sendai virus vectors [55]. Their data showed that iPS cell lines derived from the same donor were extremely similar to each other. Most of the different transcriptional, epigenetic, and differentiation propensities were donor-dependent rather than tissue source-dependent. Their data also showed the existence of variation between iPS cell lines and, more importantly, that the efficacy of reprogramming may be donor-dependent. The Hochedlinger Lab analyzed genetically matched human ES and iPS cell lines for differences in transcriptional and DNA methylation patterns [56]. They showed that variation of transcription and DNA methylation originating from genetic background dominated over variation due to cellular origin. Moreover, the 49 differentially expressed genes between genetically matched human ES and iPS cells neither predict functional outcome nor distinguish an independently derived, larger set of unmatched human ES and iPS cell lines.

These reports showed the variation between iPS cell lines. Using iPS cell lines that are almost identical to ES cells will hamper the detection of epigenetic memory of donor tissue in iPS cells, as the donor cells will have been completely reprogrammed. The detection of epigenetic memory in iPS cells depends on the degree of reprogramming.

## 8. Cancer Development through the Transient Overexpression of Reprogramming Factors

By overexpressing reprogramming factors, somatic cells acquire the properties of self-renewal along with unlimited proliferation and exhibit global alteration of the transcriptional program, which are critical events during carcinogenesis [57]. Approximately 20% of the offspring derived from retrovirally generated iPS cells developed tumors that were attributable to the reactivation of the c-Myc transgene [6]. The overexpression of Oct3/4 in somatic cells results in dysplastic growth in epithelial tissues through the inhibition of cellular differentiation in a manner similar to that in embryonic cells [58]. These studies suggest that the reprogramming processes and cancer development may be partly promoted by overlapping mechanisms [59].

Ohnishi et al. recently generated an in vivo mouse reprogramming system using reprogramming factor-inducible alleles and examined the effects of the reprogramming factor expression in somatic cells in vivo [60]. The transient expression of reprogramming factors in vivo resulted in the development of tumors consisting of undifferentiated dysplastic cells that exhibited global changes in their DNA methylation patterns in various tissues. Their data suggest that the abnormal growth of unsuccessfully reprogrammed cells predominantly depends on epigenetic regulations and that cells associated with failed reprogramming behave similarly to cancer cells.

We also recently showed the development of cancer through the transient overexpression of reprogramming factors [61]. When we generated iPS cells and iTS cells, we also generated “induced fibroblast-like (iF) cells” that were capable of self-renewal and had a similar morphology to fibroblast cells. Although the iF cells were morphologically similar to fibroblasts, they were unlikely to show adipogenic/osteogenic differentiation. Furthermore, iF cells have the ability to form tumors and behave similarly to cancer cells. The iF cells in our study may be cells in which reprogramming failed and may behave similarly to the cancer cells that arise through altered epigenetic regulation. The technology used in the generation of iPS/iTS cells is also associated with a risk of generating cancer-like cells.

## 9. Conclusions

ES/iPS cells are capable of pluripotency and ongoing proliferation. Although many iPS cells contain epigenetic memory, which is considered to be an unresolved issue with iPS cells compared with ES cells, this epigenetic memory may predispose iPS cells to differentiate more readily into their parental cells than others. The efficient differentiation potential of iPS cells with epigenetic memory may be advantageous for cell replacement therapy. iTS cells inherit numerous components of epigenetic memory from donor tissue and acquire self-renewal potential. The advantages of iTS cells over iPS cells include a more efficient differentiation and a lack of teratoma formation. The generation of iTS cells may have important implications for the clinical application of stem cells.

## Abbreviations

iPS	induced pluripotent stem
ES	embryonic stem
iTS	induced tissue-specific stem
iTS-P	iTS cells from the pancreas
iTS-L	iTS cells from the liver
DMRs	differentially methylated regions
Pdx1	pancreatic and duodenal homeobox factor-1
qRT-PCR	quantitative reverse transcription polymerase chain reaction
